# Thin Media Images Decrease Women’s Body Satisfaction: Comparisons Between Veiled Muslim Women, Christian Women and Atheist Women Regarding Trait and State Body Image

**DOI:** 10.3389/fpsyg.2019.01074

**Published:** 2019-05-10

**Authors:** Leonie Wilhelm, Andrea S. Hartmann, Julia C. Becker, Melahat Kisi, Manuel Waldorf, Silja Vocks

**Affiliations:** ^1^Department of Clinical Psychology and Psychotherapy, Osnabrück University, Osnabrück, Germany; ^2^Department of Social Psychology, Osnabrück University, Osnabrück, Germany; ^3^Department of Islamic Theology, Osnabrück University, Osnabrück, Germany

**Keywords:** state body image, trait body image, thin media images, veiling, religiosity, Muslim women

## Abstract

Research in diverse populations has often found that thin media images negatively affect women’s state body image, with many women reporting lower body satisfaction after exposure to pictures of thin models than before exposure. However, there is evidence that theistic affirmations might buffer against the negative effect of media on body image. Furthermore, religiosity and the Islamic body covering are discussed as protective factors against a negative trait body image. However, there is no experimental research on veiled Muslim women’s state body image. Therefore, the current study experimentally investigated whether the body satisfaction of veiled Muslim women (*n* = 66) decreased after exposure to thin media images compared to pictures of furniture as a control condition. Christian women (*n* = 90) and atheist women (*n* = 74) were included as control groups, and participants were randomly assigned to the two conditions. Prior to the experimental session, participants’ trait body image was assessed using an online questionnaire comprising questions about body satisfaction, thin-ideal internalization, pressure to be thin, and physical appearance comparisons. It was found that veiled Muslim women had a more positive trait body image than did Christian women and atheist women. Accordingly, veiled Muslim women reported lower levels of thin-ideal internalization, pressure to be thin, and physical appearance comparisons than did Christian women and atheist women. The experimental findings showed that body satisfaction decreased in the experimental condition and not in the control condition, but no significant differences in pre-post changes emerged between the three groups. As the pre-post changes in body satisfaction did not differ between the three groups, veiling might not buffer against the negative effect of thin media images on state body image. Nevertheless, given the more positive trait body image of veiled Muslim women compared to Christian and atheist women, veiling might positively influence body image in the longer term. However, as additional analyses including unveiled Muslim women did not reveal differences between veiled and unveiled Muslim women, future studies should test the assumption that affiliation to Islam might be more decisive for a positive trait body image than veiling.

## Introduction

According to Slade (1994), body image is defined as the individual mental representation of one’s own body and the feelings one has regarding this representation. Besides these perceptions and feelings, body image encompasses two further components: the cognitive component, including body-related thoughts, and the behavioral component (Cash, 2004). The latter includes avoidance behaviors, e.g., not looking in the mirror, and body-checking behaviors, e.g., weighing oneself or scrutinizing one’s own body weight, size or shape (Hartmann et al., 2019). Moreover, it consists of a cross-situational aspect, the trait body image, and a more variable aspect, the state body image (Cash et al., 2002; Rudiger et al., 2007). The latter is more easily influenced by external stimuli, such as the intake of high-calorie food (Vocks et al., 2007), physical exercise (Vocks et al., 2009), or the presentation of thin models (Legenbauer et al., 2008). The permanent confrontation with ultra-thin models in the mass media is assumed to be one key factor in the development of a negative body image (Thompson and Heinberg, 1999). Therefore, many experimental studies (see the following meta-analyses: Groesz et al., 2002; Grabe et al., 2008; Want, 2009; Ferguson, 2013) have examined how different stimuli, for instance pictures of thin, average-weight, or overweight models as opposed to pictures of cars and furniture, influence the state body image of various populations. As most research on body image is based on cross-sectional or correlational designs, experimental studies are highly important in this context. In many of these studies, participants reported a more negative state body image and a greater negative affect after exposure to thin images compared to pictures of average-weight models or furniture. However, it is important to note that only some participants were negatively affected by thin-ideal stimuli, while the majority were not (Ferguson, 2013). Given that a negative body image is presumed to be a risk factor for the development of eating disorders (Stice and Shaw, 2002; Fiske et al., 2014), it is relevant to examine potential factors that might buffer against negative effects of media. In this context, factors like ethnicity or religiosity have been discussed (Ferguson, 2013; Tiggemann, 2015).

For instance, trait body image was found to positively correlate with religiosity and a positive, secure, and intrinsically motivated relationship with God (Forthun et al., 2003; Homan and Boyatzis, 2010; Homan and Cavanaugh, 2013; Akrawi et al., 2015). Furthermore, a review concluded that strong religious beliefs and a satisfying relationship with God are negatively associated with disordered eating and body image concerns in Christian and Jewish women (Akrawi et al., 2015). In contrast, in a sample of Muslim college women from the United Arab Emirates, a positive relationship was found between extent of religiosity and eating disorder symptoms (Thomas et al., 2018). However, other studies failed to find differences in disordered eating between Muslim women with higher and lower levels of religiosity (Wilhelm et al., 2018) or between Christian female college students with and without strong spiritual and religious beliefs (Jacobs-Pilipski et al., 2005). Possible reasons for the contradictory findings might lie in the different types of samples, the individual relationship to God, or the various religious affiliations.

With respect to Islam, a recently growing number of studies has investigated the trait body image of Muslim women (e.g., Demmrich et al., 2017; Kertechian and Swami, 2017; Wilhelm et al., 2018). The results indicated that modesty of clothing mediated the negative relationships between Muslim women’s extent of religiosity and body dissatisfaction as well as dietary behavior (Mussap, 2009). Furthermore, when controlling for religiosity, British Muslim women who wore the Islamic headscarf, often called the hijab, reported a lower internalization of the standard of beauty and a lower pressure to conform to this standard than those who did not wear the hijab (Swami et al., 2014). Therefore, when investigating body image in Muslim women, it is important to distinguish between Muslim women who veil their bodies and Muslim women who do not veil (Demmrich et al., 2017; Kertechian and Swami, 2017). Compared to unveiled Muslim women, veiled Muslim women were found to appreciate their bodies more (Swami et al., 2014), reported lower levels of body dissatisfaction and drive for thinness (Swami et al., 2014; Kertechian and Swami, 2017), and showed less body checking behavior (Wilhelm et al., 2018). Moreover, compared to unveiled Turkish Muslim women, veiled women less often feared negative evaluations from others regarding their physical appearance (Demmrich et al., 2017). These results suggest that, when controlling for religiosity, veiling might buffer against a negative body image. However, veiled and unveiled Canadian Muslim women were as satisfied with their appearance and weight as were non-Muslim women, and reported similar levels of thin-ideal internalization as well as pressure to conform to the standards of beauty which are transported by the media (Chaker et al., 2015). Furthermore, veiled and unveiled Australian Muslim women did not differ from non-Muslim women in terms of body dissatisfaction, dietary behavior, and media consumption (Mussap, 2009). Thus, although veiled Muslim women partly show a more positive trait body image than do unveiled and non-Muslim women, the Islamic body covering is not generally linked to a positive body image. Possible explanations for the discrepant results might lie in the age at which a woman begins to cover her body (Kertechian and Swami, 2017), the duration of veiling over a day, or the country in which a woman lives (Tiggemann, 2015).

Besides this questionnaire-based research assessing trait body image, several experimental studies have investigated whether religious beliefs or the attachment to God might protect women’s state body image against negative effects of media (Boyatzis et al., 2007; Homan, 2012; Inman et al., 2014). Christian college women with a secure attachment to God reported a more positive state body image following exposure to images of ultra-thin models than did women with a less secure attachment to God (Homan, 2012). Furthermore, Christian college women who read body-related theistic affirmations before being presented with pictures of thin models were more satisfied with their appearance compared to women who had read neutral news (Boyatzis et al., 2007). However, the two groups did not differ regarding weight satisfaction after the exposure. Moreover, Inman et al. (2014) were unable to replicate Boyatzis et al.’s (2007) finding that theistic affirmation led to a greater appearance satisfaction, but did replicate the null findings regarding satisfaction with weight. In view of these findings, the reading of theistic affirmations before exposure to pictures of thin models might not be sufficient to buffer all facets of women’s body image against the effects of thin media images.

In this context, it is important to note that existing experimental research on religiosity and state body image has often excluded non-religious women and mainly focused on Christian women (e.g., Boyatzis et al., 2007; Inman et al., 2014). Therefore, it is of interest to compare differences in state body image not only between women with higher and lower levels of religiosity, but also between non-religious women and women of different religious affiliations. For example, although the number of Muslim persons is increasing worldwide (Lipka and Hackett, 2015), and the results of questionnaire-based studies suggest that, in part, veiled Muslim women have a more positive trait body image than do unveiled women, there is not yet any experimental research on the state body image of veiled Muslim women.

Therefore, the current two-part study assessed participants’ trait body image using an online questionnaire (Part 1) and experimentally investigated, for the first time, whether veiled Muslim women’s state body image is affected by exposure to thin media images (Part 2). Based on recent findings (Swami et al., 2014; Demmrich et al., 2017; Kertechian and Swami, 2017), it was hypothesized that veiled Muslim women would report a more positive trait body image compared to Christian women and atheist women (Hypothesis 1). In particular, it was expected that veiled Muslim women would report lower levels of eating, weight, and shape concerns, drive for thinness, body dissatisfaction, thin-ideal internalization, and pressure to be thin. Furthermore, it was hypothesized that veiled Muslim women would report fewer physical appearance comparisons than Christian women and atheist women. In line with previous findings (Groesz et al., 2002; Want, 2009), it was assumed that women would report a more negative state body image after watching a slide show of thin models compared to viewing pictures of furniture (Hypothesis 2). In this context, it was expected that after exposure to thin media images, the decrease in state body satisfaction of veiled Muslim women would be lower than that of Christian women and atheist women (Hypothesis 3), as studies suggest a protective effect of the hijab (Mussap, 2009; Swami et al., 2014; Kertechian and Swami, 2017).

## Materials and Methods

### Participants

In total, *N* = 289 participants completed the online questionnaire and *n* = 248 (85.8%) of these also attended and completed the experimental session. Inclusion criteria were female sex and an age of at least 16 years. Furthermore, participants had to self-define as Muslims, Christians or atheist; thus, females from other religious affiliations (*n* = 3) were excluded from the present study. Furthermore, *n* = 1 veiled and *n* = 1 unveiled Muslim women were excluded due to missing data. Given that previous research on body image in Muslim women differentiated between Muslim women who never veil and Muslim women who veil at least occasionally (Swami et al., 2014; Kertechian and Swami, 2017), the same criteria were used in the present study, as this allows for comparison of the current results with findings from these studies. However, in the current study, only *n* = 13 Muslim women reported never wearing any kind of Islamic body covering. Therefore, the group of unveiled Muslim women was excluded from the group comparisons, but descriptive results for unveiled Muslim women are provided as supplementary material. Moreover, all analyses were repeated with a merged group of Muslim women consisting of veiled and unveiled Muslim women. Thus, the final sample for the main data analysis consisted of *n* = 230 women. Participants were classified according to their religious affiliation. Therefore, three groups were formed, consisting of *n* = 66 Muslim women who wore some kind of Islamic body covering at least occasionally, *n* = 90 Christian women, and *n* = 74 atheist women. According to a-priori calculations with α = 0.05 and power set at 0.85, the sample size met the requirements for detecting a medium effect (G^∗^Power; Cohen, 1988; Faul et al., 2007).

### Part 1

#### Hijab Index

The ‘hijab index’ by Tolaymat and Moradi (2011), which comprises a frequency and conservativeness dimension, was used to assess whether and how Muslim women cover their bodies in public. Hijab frequency [“How frequently do you wear an Islamic headscarf (e.g., hijab, chador, burqa, etc.)?”] was rated on a 5-point scale from 1 (*never*) to 5 (*always*). Conservativeness was rated on a 7-point scale from 0 (*no covering*) to 6 (*long covering from head to feet and face covered completely*), with response options depicted by six drawings displaying various styles of hijab complemented by written descriptions. Hijab frequency and hijab conservativeness were multiplied to form a hijab index score, with a possible range from 0 to 30. In accordance with Tolaymat and Moradi (2011), participants also reported their reasons for wearing the hijab, with various options being provided based on previous literature. It is possible to select multiple reasons and to add a non-listed reason. Additionally, participants reported the age at which they began to cover their bodies and indicated the number of hours for which they usually cover their bodies on an average day.

#### Centrality of Religiosity Scale

The Centrality of Religiosity Scale (Huber and Huber, 2012), which was originally created to assess the extent of religiosity in Christian samples, was used as it offers an adaptation for Muslim samples. The 15-item self-report measure (e.g., “How often do you think about religious issues?”) is rated on a 5-point Likert scale ranging from 1 (*never/not at all*) to 5 (*very often/very much so*). The scale was used to assess whether and how participants practice a religion and to determine how devout they are. According to their Centrality of Religiosity Scale scores, persons can be classified as “highly religious” (5.0–4.0), “religious” (3.9–2.1), and “non-religious” (2.0–1.0; Huber and Huber, 2012). The authors reported excellent reliabilities for the scale, with Cronbach’s alpha ranging from α = 0.92 to α = 0.96. In the present study, Cronbach’s alpha amounted to α = 0.98.

#### Media Exposure

In accordance with previous research on body image (e.g., Mussap, 2009), a self-report measure of media exposure (van den Berg et al., 2007) was used to calculate participants’ extent of media consumption. The measure consists of two items assessing frequency of TV- and video-viewing and one item rating exposure to magazines with dieting-related messages. Participants had to indicate hours of TV- and video-viewing on a 7-point scale from 1 (0 h) to 7 (5+ h) twice; first for an average weekday and second for an average weekend day. In order to determine the total hours of TV- and video-viewing in an average week, a weighted sum of participants’ responses was calculated. Magazine message exposure (“How often do you read magazine articles in which dieting or weight loss are discussed?”) was rated on a 4-point scale from 1 (*never*) to 4 (*often*).

#### Eating Disorder Examination-Questionnaire

The self-report Eating Disorder Examination-Questionnaire (Fairburn and Beglin, 1994; German-language version: Hilbert and Tuschen-Caffier, 2016) was used in order to assess the main features of eating disorders and their intensity and frequency during the past 28 days. The questionnaire consists of four subscales: *Restraint* (5 items), *Eating Concerns* (5 items), *Shape Concerns* (8 items), and *Weight Concerns* (5 items). The 23 items were rated on a 7-point Likert scale ranging from 0 (*no days/not at all*) to 6 (*every day/markedly*). With Cronbach’s alphas ranging from α = 0.85 to α = 0.97 (Hilbert et al., 2007), the questionnaire has proven to be internally consistent. In the current study, Cronbach’s alpha ranged from α = 0.76 to α = 0.94 for the total score and the four subscales.

#### Eating Disorder Inventory 2

The three subscales *Drive for thinness*, *Bulimia*, and *Body dissatisfaction* from the Eating Disorder Inventory 2 (Garner et al., 1983; German-language version: Rathner and Waldherr, 1997) were used in the present study. Each item was rated on a 6-point Likert scale ranging from 1 (*never*) to 6 (*always*). The 7-item subscale *Drive for Thinness* assesses an individual’s urge to be thin, whereas the 7-item subscale *Bulimia* measures psychopathological eating. The 9-item subscale *Body Dissatisfaction* assesses dissatisfaction with the size and shape of one’s body parts. The authors reported internal consistencies ranging from α = 0.73 to α = 0.93 for the three subscales. In the current study, Cronbach’s alpha ranged from α = 0.84 to α = 0.91.

#### Sociocultural Attitudes Toward Appearance Questionnaire

The self-report Sociocultural Attitudes Toward Appearance Questionnaire (Heinberg et al., 1995; German-language version: Knauss et al., 2009) was used to assess the influence of media body ideals on body image. The 16 items are divided into three subscales: *Internalization of Media Body Ideals* (6 items), *Perceived Pressure from Media* (5 items), and *Awareness of the Body Ideal* (5 items). Each item was rated on a 5-point Likert scale ranging from 1 (*completely disagree*) to 5 (*completely agree*). The authors reported internal consistencies ranging from α = 0.75 to α = 0.89 for the three subscales. In the present study, Cronbach’s alpha ranged from α = 0.81 to α = 0.92.

#### Physical Appearance Comparison Scale

In order to assess how frequently participants, compare their own physical appearance with that of others, the self-report *Physical Appearance Comparison Scale* (Thompson et al., 1991; German-language version: Berger et al., 2009) was used. The scale consists of 5 items, which are rated on a 5-point Likert scale ranging from 1 (*never*) to 5 (*always*). Internal consistency lies at α = 0.78. In the present study, Cronbach’s alpha amounted to α = 0.79.

### Part 2

#### Body Image States Scale

The 6-item self-report Body Image States Scale (Cash et al., 2002; German-language version: Vocks et al., 2009) was used to calculate the effect of thin media images on participants’ body image. In order to assess the state body image, the items begin with “Right now I feel…” and are answered on a scale from 1 (*extremely dissatisfied*) to 9 (*extremely satisfied*). Good internal consistencies were found for the scale, with Cronbach’s alpha ranging from α = 0.77 to α = 0.90 (Cash et al., 2002). In the present study, Cronbach’s alpha amounted to α = 0.90 and α = 0.92 for the pre- and post-score, respectively.

#### Positive and Negative Affect Schedule

The self-report Positive and Negative Affect Schedule (Watson et al., 1988; German-language version: Krohne et al., 1996) was used to measure the effect of thin media images on participants’ mood. It is divided into two subscales, with 10 items assessing positive and 10 items assessing negative affective states on a scale from 1 (*very slightly or not at all*) to 5 (*extremely*). For the “Moment” time instruction of the scale assessing *Negative Affective States*, which was used in the present study, the authors reported an internal consistency of α = 0.86. Cronbach’s alpha for the pre- and post-assessment in the current study amounted to α = 0.82 and α = 0.85, respectively.

#### Emotions Toward the Models

To assess emotions that participants in the experimental condition might have toward the presented models, the women were asked to indicate the degree to which they feel admiration, rage, contempt, and disgust toward the presented models. Each emotion was rated on a scale from 1 (*not at all*) to 4 (*extremely*).

#### Stimulus Material

In line with previous studies (see meta-analysis by Grabe et al., 2008), full-body pictures of thin and attractive models wearing short and tight clothes were presented in the experimental condition, whereas neutral pictures displaying furniture were shown in the control condition (all pictures were bought from a stock photography agency). As in similar research (Harper and Tiggemann, 2008; Homan, 2012), the model pictures were selected by 30 independent females who did not participate in the study and who rated the attractiveness and thinness of 26 models on a scale from 1 (*not at all attractive/thin*) to 7 (*extremely attractive/thin*). In order to use pictures that represent the type of models currently seen in Germany, the models were unveiled, but varied with respect to ethnicity and color of the skin, hair, and eyes. Moreover, in a small pilot test, one of the co-authors, a veiled Muslim, surveyed friends from the target group with regard to relevance of the pictures to them. In their opinion, the pictures were appropriate for also triggering the state body image of veiled Muslim women, as was intended by the design of the current study. As a meta-analysis (Want, 2009) stated that higher effect sizes are generated by presenting at least 11 stimuli and by reducing the exposure time to a maximum of 5 min, the 20 best-rated pictures were chosen and each picture was presented for 15 s. All selected models were evaluated as attractive (*M* = 4.92, *SD* = 0.36) and very thin (*M* = 5.48, *SD* = 0.45).

### Procedure

The present study was carried out in accordance with the Declaration of Helsinki and the recommendations of the Ethics Committee of Osnabrück University. The protocol was approved by the Ethics Committee of Osnabrück University. All subjects gave written informed consent in accordance with the Declaration of Helsinki. The present study consisted of two parts. The first part was an online survey including the trait measures, which participants completed at home. The second part consisted of a computer-based experiment, which took place at the laboratory of the university. To prevent an influence of completing the trait questionnaires on responses to the state measures, the online survey had to be completed at least 48h before the experimental session. Participants were recruited through different mailing lists (e.g., universities, Muslim associations), advertisements on Facebook, personal conversations, and placards in University buildings. Potential participants were told that the study was about body image; thus, no cover story was used. After participants expressed their interest via email, they were informed about the procedure and were provided with the link to the online survey, which was designed using EFS survey (Questback GmbH, Cologne, Germany).

#### Part 1

On the first page of the survey, participants were asked to read and agree to the terms and conditions of data privacy protection. They assigned themselves an individual code, which they were also asked to provide in the experimental session, enabling both parts of the study to be merged. Then, participants answered demographic questions (e.g., religious affiliation) before being presented with the aforementioned trait questionnaires. In total, the survey took an average of 25 min to complete. On the last page, participants were thanked and were informed that they must also participate in the experiment in order to receive their compensation consisting of course credits or cash (5€).

#### Part 2

Participants were randomly assigned to the experimental (*n* = 118) or the control condition (*n* = 112). The experiment was presented on 23-inch screens (Dell^TM^ OptiPlex^TM^ 780). In both conditions, participants sat approximately 50 cm away from the screen. They were asked to read and sign the consent form, before starting the experiment by pressing any button on the keyboard. All instructions were presented on the screen. First, participants completed the state questionnaires, before being presented either with pictures of thin models in the experimental condition or with pictures of furniture in the control condition. After viewing the thin media images, participants in the experimental condition had to indicate their emotions toward the presented models, and participants in both conditions again completed the two state questionnaires. Finally, participants received their reward. The Ethics Committee of the University approved the study.

### Statistical Analysis

The data analysis was conducted using the Statistical Package for the Social Sciences SPSS 24 (IBM; Armonk, United States). First, group comparisons were conducted between veiled Muslim women, Christian women, and atheist women, and descriptive data were calculated including unveiled Muslim women as a separate group. In a second step, all comparisons between the three groups and the correlations within the Muslim group were repeated with a merged group of Muslim women that consisted of veiled and unveiled Muslim women. The results of these additional analyses were reported only in the case of any significant deviation from the first results. In general, in the case of significant results, *post hoc* comparisons including Bonferroni adjustment were conducted. The level of significance was set at *p* < 0.05 (two-tailed), which was adjusted in the case of multiple comparisons, and partial η^2^ or Cohen’s *d* were used to determine effect sizes. In accordance with conventional classifications, η^2^ = 0.01 and *d* = 0.2 indicate small, η^2^ = 0.06 and *d* = 0.5 indicate medium, and η^2^ = 0.12 and *d* = 0.8 indicate large effects, respectively (Cohen, 1988; Lakens, 2013).

#### Part 1

First, univariate analyses and χ^2^-tests were conducted to test whether the three groups differed in self-reported body mass index (BMI; kg/m^2^), age, country of birth, relationship status, educational background, Centrality of Religiosity Scale scores, and media consumption. Second, means and frequency analyses were conducted for the hijab index in order to determine since when, for how long, and why veiled Muslim women cover their bodies. Additionally, Pearson product-moment correlations were run on all trait body image measures and the hijab index as well as on all trait body image measures and the age at which a woman began to veil. Third, multivariate (MANCOVA) and univariate analyses of covariance (ANCOVA) were calculated including BMI as a covariate. Three separate MANCOVAs were conducted to compare group scores on the Eating Disorder Examination-Questionnaire subscales, the Eating Disorder Inventory 2 subscales, and the Sociocultural Attitudes Toward Appearance Questionnaire scales. In order to compare group scores on the Physical Appearance Comparison Scale, an ANCOVA was calculated.

#### Part 2

A three-way 2 × 2 × 3 repeated measures univariate analysis of covariance (rmANCOVA) was conducted with Time (pre, post) as within-subject factor and Condition (experimental, control) and Group (Muslim, Christian, atheist) as between-subject factors, respectively. In order to investigate whether changes in state body image are linked to participants’ existing body image concerns, pre-exposure scores on the Body Image States Scale were subtracted from post-exposure scores and the resulting pre-to-post scores were correlated with various trait measures in the experimental condition. Positive scores indicate an increase in body satisfaction from pre- to post-exposure, whereas negative scores display a decrease.

## Results

### Participants

Significant group differences were found regarding BMI, country of birth, and relationship status (see Table 1). Muslim women had a higher BMI than did Christian women, *p* < 0.001, *d* = 0.61, and atheist women, *p* < 0.001, *d* = 0.67. Regarding country of birth, veiled Muslim women were less often born in Germany than were Christian women, *p* < 0.001, *d* = 0.72, and atheist women, *p* = 0.002, *d* = 0.54. Compared to Christian women, *p* < 0.001, *d* = 0.60, and atheist women, *p* = 0.003, *d* = 0.53, veiled Muslim women less often reported being in a current relationship.

**Table 1:** Groups’ characteristics for body mass index, age, country of birth, relationship status, educational level, Centrality of Religiosity Scale scores, and media consumption (*N* = 230).

Variable	Possible range	Muslim women (*n* = 66)		Christian women (*n* = 90)		Atheist women (*n* = 74)		Group comparison			
		*M*	*SD*	*M*	*SD*	*M*	*SD*	*F*	*df*	*P*	ηp^2^
Body mass index		24.05^a,b^	4.43	21.65^c^	3.51	21.68^c^	2.49	10.752^∗∗^	2	<0.001	0.09
Age		22.11	4.05	21.29	3.07	21.73	3.97	0.840	2	0.433	0.01
*Number of participants*											
Born in Germany		52 (78.8%)^a,b^		89 (98.9%)^c^		71 (96.0%)^c^		χ^2^ = 23.046^∗∗^		<0.001	0.10
Being in a current relationship		15 (22.7%)^a,b^		46 (51.1%)^c^		35 (47.3%)^c^		χ^2^ = 14.521^∗^		0.001	0.06
With university degree		7 (10.4%)		7 (7.8%)		6 (8.1%)		χ^2^ = 0.388		0.824	0.00
Centrality of Religiosity Scale	1–5	4.42^a,b^	0.40	2.64^b,c^	0.68	1.68^a,c^	0.39	497.291^∗∗^	2	<0.001	0.81
*Media Consumption*											
TV- and video-viewing hours	1–7	2.57	1.57	2.38	1.34	2.50	1.50	0.317	2	0.729	0.00
Reading of dieting articles	1–4	1.80	0.77	2.00	0.72	1.85	0.68	1.630	2	0.198	0.01

*^∗∗^p < 0.001; ^∗^p < 0.05. ^a^Differ significantly from Christian women (p < 0.05). ^b^Differ significantly from atheist women (p < 0.05). ^c^Differ significantly from Muslim women (p < 0.05).*

### Part 1

#### Centrality of Religiosity Scale

The ANOVA yielded significant results (see Table 1), with subsequent *post hoc* tests revealing that veiled Muslim women reported greater scores than Christian women, *p* < 0.001, *d* = 3.17, and atheist women, *p* < 0.001, *d* = 6.97. Furthermore, Christian women scored higher than atheist women, *p* < 0.001, *d* = 1.69.

#### Hijab Index

The hijab index yielded an average of *M* = 13.36 (*SD* = 4.03). Veiled Muslim women started to veil at an average age of *M* = 14.92 (*SD* = 4.06). The year in which they began to veil was significantly correlated with BMI, *r* = 0.287, *p* = 0.020, and with scores on the Physical Appearance Comparison Scale, *r* = −0.244, *p* = 0.048. The veiled Muslim women reported covering their bodies for *M* = 8.09 (*SD* = 2.70) hours over the course of a day. Significant correlations were found between frequency of veiling and conservativeness of the covering, *r* = 0.386, *p* = 0.001, and between the hijab index and participants’ extent of religiosity, *r* = 0.525, *p* < 0.001. When asked why they practice the Islamic body covering, most women (98.5%) stated “religious reasons”, 65.2% stated “to protect my body against the view of others”, 30.3% stated “to show others my Muslim identity”, and 30.3% stated “to feel comfortable in mixed-sex settings”. Finally, 10.6% mentioned “to protect against Western ideas or lifestyle” as a reason for their veiling. With regard to body image variables, the hijab index was significantly correlated with *Internalization of media body ideals*, *r* = −0.320, *p* = 0.009, *Awareness of the thin ideal*, *r* = −0.370, *p* = 0.002, and the score on the Physical Appearance Comparison Scale, *r* = −0.310, *p* = 0.011. No significant correlations were found between the hijab index and participants’ BMI, eating, weight, and shape concerns, body dissatisfaction, drive for thinness, as well as perceived pressure from the media. The negative correlations between the hijab index and *Internalization of media body ideals*, *Awareness of the thin ideal*, and the score on the Physical Appearance Comparison Scale did not reach significance when the merged group of veiled and unveiled Muslim women was included in the analysis.

#### Eating Disorder Examination-Questionnaire

The results of the MANCOVA for the four subscales turned out to be significant, Wilks’ λ = 0.928, *F*(8,446) = 2.122, *p* = 0.033, η_p_*^2^* = 0.04. Subsequent ANCOVAS including *post hoc* tests revealed that veiled Muslim women showed fewer shape concerns than did Christian women, *p* = 0.015, *d* = 0.24, and atheist women, *p* = 0.008, *d* = 0.29, and reported lower levels of weight concerns than did atheist women, *p* = 0.029, *d* = 0.17 (see Table 2).

**Table 2:** Means (*M*) and standard deviations (*SD*) of the Eating Disorder Examination Questionnaire, the Eating Disorder Inventory 2 subscales, the Physical Appearance Comparison Scale, the Sociocultural Attitudes Toward Appearance Questionnaire, and the emotions toward the models (*N* = 230).

Variable	Possible range	Muslim women (*n* = 66)		Christian women (*n* = 90)		Atheist women (*n* = 74)		ANCOVA with Bonferroni-adjusted *post hoc* tests			
		*M*	*SD*	*M*	*SD*	*M*	*SD*	*F*	*df*	*P*	ηp^2^
*Eating Disorder Examination Questionnaire*											
Restraint	0–6	0.95	1.07	1.32	1.30	1.14	1.19	2.423	2	0.091	0.02
Eating Concerns	0–6	0.75	0.95	0.78	0.84	0.84	0.92	1.072	2	0.344	0.01
Shape Concerns	0–6	1.79^a,b^	1.46	2.13^c^	1.44	2.20^c^	1.40	5.440^∗^	2	0.005	0.05
Weight Concerns	0–6	1.60^b^	1.44	1.70	1.36	1.84^c^	1.37	3.661^∗^	2	0.027	0.03
*Eating Disorder Inventory 2*											
Drive for thinness	1–6	2.91	1.13	3.01	1.27	3.13	1.29	2.999	2	0.052	0.03
Bulimia	1–6	1.74	0.70	1.73	0.60	1.88	0.81	2.751	2	0.066	0.02
Body dissatisfaction	1–6	3.16^a.b^	1.34	3.24^c^	1.14	3.37^c^	1.05	5.072^∗^	2	0.007	0.04
*Sociocultural Attitudes Toward Appearance Questionnaire*											
Internalization	6–30	12.11^a,b^	5.88	15.57^c^	5.70	15.70^c^	6.38	9.520^∗∗^	2	0.000	0.08
Pressure	5–25	10.58^a,b^	5.57	12.60^c^	4.64	12.73^c^	5.42	4.331^∗^	2	0.014	0.04
Awareness	5–25	15.17	4.50	15.54	3.61	16.49	3.79	2.642	2	0.073	0.02
Physical Appearance Comparison Scale	5–25	11.20^a,b^	4.29	15.06^c^	3.59	14.04^c^	3.49	20.295^∗∗^	2	<0.001	0.15
*Emotions toward the models^d^*											
Admiration	1–4	1.59	0.82	1.89	0.37	1.89	0.71	1.928	2	0.150	0.03
Rage	1–4	1.15	0.36	1.20	0.40	1.25	0.55	0.472	2	0.625	0.01
Contempt	1–4	1.24	0.61	1.28	0.50	1.17	0.38	0.540	2	0.584	0.01
Disgust	1–4	1.32	0.59	1.30	0.47	1.19	0.47	0.692	2	0.502	0.01

*^∗∗^p < 0.001; ^∗^p < 0.05. ^a^Differ significantly from Christian women (p < 0.05). ^b^Differ significantly from atheist women (p < 0.05). ^c^Differ significantly from Muslim women (p < 0.05). ^d^Emotions toward the models were only assessed in the experimental condition.*

#### Eating Disorder Inventory 2

For the three subscales, the MANCOVA failed to reach statistical significance, Wilks’ λ = 0.948, *F*(6,448) = 2.004, *p* = 0.064, η_p_*^2^* = 0.03, indicating no significant differences between the three groups. Table 2 displays the group means and the results of the univariate analyses. In the additional analyses regarding the merged group of veiled and unveiled Muslim women, the MANCOVA turned out to be significant, Wilks’ λ = 0.943, *F*(6,474) = 2.355, *p* = 0.030, η_p_*^2^* = 0.03. Subsequent ANCOVAS indicated that the merged group of veiled and unveiled Muslim women differed from Christian women as well as atheist women regarding body dissatisfaction, *p* = 0.003, drive for thinness, *p* = 0.028, and bulimia symptoms, *p* = 0.031. Subsequent *post hoc* tests revealed that the merged group of veiled and unveiled Muslim women reported lower levels of body dissatisfaction (*M* = 3.10, *SD* = 1.34) than did Christian women, *p* = 0.025, *d* = 0.11, and atheist women, *p* = 0.004, *d* = 0.22. Furthermore, compared to atheist women, the merged group of veiled and unveiled Muslim women reported lower levels of drive for thinness (*M* = 2.87, *SD* = 1.16), *p* = 0.033, *d* = 0.21, as well as bulimia symptoms (*M* = 1.69, *SD* = 0.67), *p* = 0.026, *d* = 0.26.

#### Sociocultural Attitudes Toward Appearance Questionnaire.

The MANCOVA for the three subscales yielded significant differences between the three groups, Wilks’ λ = 0.911, *F*(6,448) = 3.568, *p* = 0.002, η_p_*^2^* = 0.05. Subsequent ANCOVAS including *post hoc* tests indicated that veiled Muslim women reported lower scores on *Internalization of Media Body Ideals* than did Christian women, *p* < 0.001, *d* = 0.60, and atheist women, *p* < 0.001, *d* = 0.58. Furthermore, they scored lower on *Perceived Pressure from Media* than did Christian women, *p* = 0.030, *d* = 0.40, and atheist women, *p* = 0.027, *d* = 0.39 (see Table 2). In the additional analyses regarding the merged group of veiled and unveiled Muslim women, the merged group also reported lower levels of *Awareness* (*M* = 14.94, *SD* = 4.44) than did atheist women, *p* = 0.019, *d* = 0.38.

#### Physical Appearance Comparison Scale

The ANCOVA yielded significant group differences regarding the scores on the Physical Appearance Comparison Scale (see Table 2), with subsequent *post hoc* tests indicating that Muslim women reported lower levels than did Christian women, *p* < 0.001, *d* = 0.99, and atheist women, *p* < 0.001, *d* = 0.73.

### Part 2

#### Body Image States Scale

**FIGURE 1:**
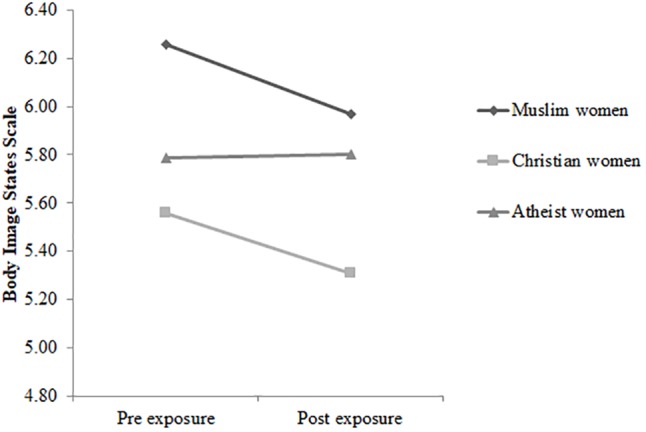
The time course of scores on the Body Image States Scale for veiled Muslim women (*n* = 34), Christian women (*n* = 48), and atheist women (*n* = 36) in the experimental condition.

**FIGURE 2:**
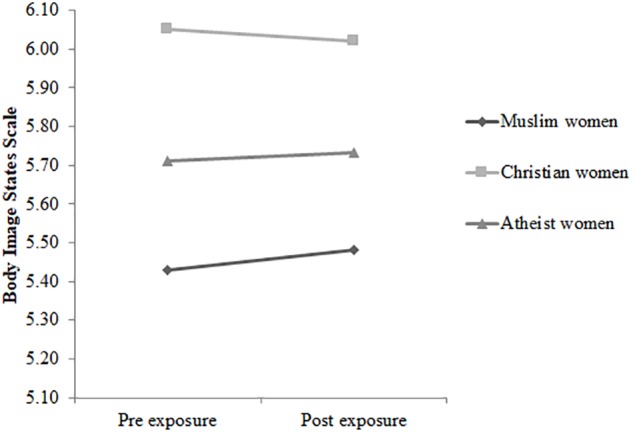
The time course of scores on the Body Image States Scale for veiled Muslim women (*n* = 32), Christian women (*n* = 42), and atheist women (*n* = 38) in the control condition.

In terms of the pre-exposure scores, no significant group differences were found, *p* = 0.901. The three-way rmANCOVA Time × Condition × Group did not reach significance, Wilks’ λ = 0.982, *F*(2, 217) = 2.04, *p* = 0.132, η_p_*^2^* = 0.02. However, the interaction Time × Condition turned out to be significant, *F*(1,217) = 8.034, *p* = 0.005, η_p_*^2^* = 0.04, with *post hoc* tests revealing that body satisfaction declined in the experimental condition (see Figure 1), *p* < 0.001, *d* = 0.36, but did not change in the control condition (see Figure 2), *p* = 0.758, *d* = 0.04. Furthermore, the interaction Condition × Group just reached the significance level, *F*(2,217) = 3.04, *p* < 0.05, η_p_*^2^* = 0.03. Subsequent *post hoc* tests indicated that in the experimental condition, veiled Muslim women reported greater levels of body satisfaction than did Christian women before, *p* = 0.003, *d* = 0.49, and after the exposure, *p* = 0.012, *d* = 0.40. In the additional analyses regarding the merged group of veiled and unveiled Muslim women, the interaction Condition × Group did not reach significance. Participants’ means are displayed in Table 3.

**Table 3:** Means (*M*) and Standard deviations (*SD*) of the Body Image States Scale (BISS) and the subscale negative affective states (NAS) of the Positive and Negative Affect Schedule for the experimental (*N* = 118) and the control condition (*N* = 112).

Variable	Possible range	Time	Muslim women		Christian women		Atheist women	
			*M*	*SD*	*M*	*SD*	*M*	*SD*
*Experimental condition*			(*n* = 34)		(*n* = 48)		(*n* = 36)	
BISS	1–9	Pre	6.26^a^	1.39	5.56^c^	1.45	5.79	1.09
	1–9	Post	5.97^a^	1.76	5.3^c^	1.60	5.80	1.17
NAS	10–50	Pre	12.74	3.11	13.83	5.77	13.25	3.50
	10–50	Post	13.00	3.80	14.40	6.43	12.92	3.22
*Control condition*^d^			(*n* = 32)		(*n* = 42)		(*n* = 38)	
BISS	1–9	Pre	5.43	1.91	6.05	1.17	5.71	1.38
	1–9	Post	5.48	1.97	6.02	1.16	5.73	1.40
NAS	10–50	Pre	14.75	4.45	12.93	3.03	13.76	4.01
	10–50	Post	14.31	4.67	11.95	2.47	12.47	3.54

*^∗∗^p < 0.001; ^∗^p < 0.05. ^a^differ significantly from Christian women (p < 0.05). ^b^differ significantly from atheist women (p < 0.05). ^c^differ significantly from Muslim women (p < 0.05). ^d^Body Image States Scale scores were not recorded for three Christian women and three atheist women in the control condition.*

#### Positive and Negative Affect Schedule

**FIGURE 3:**
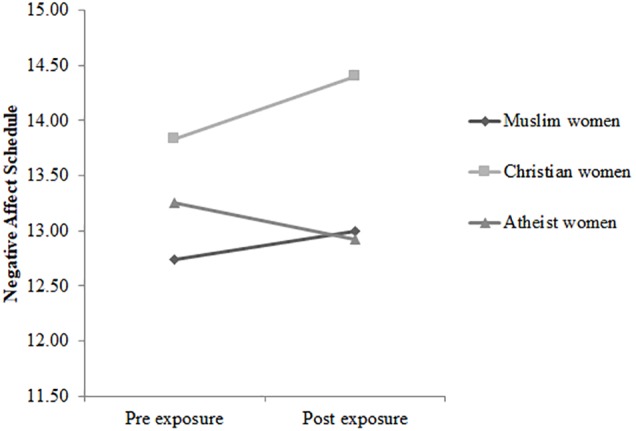
The time course of scores on the Negative Affect Schedule for veiled Muslim women (*n* = 34), Christian women (*n* = 48), and atheist women (*n* = 36) in the experimental condition.

**FIGURE 4:**
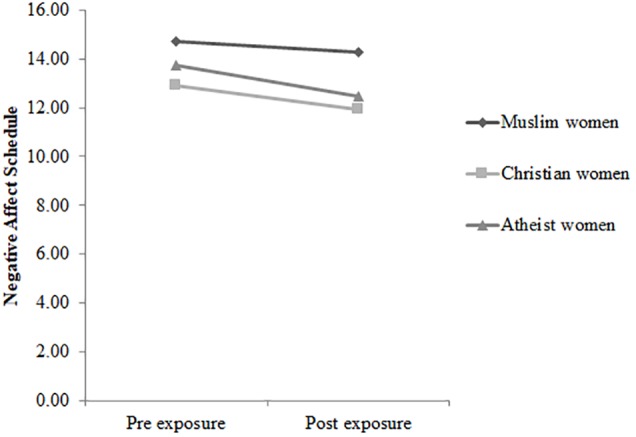
The time course of scores on the Negative Affect Schedule for veiled Muslim women (*n* = 32), Christian women (*n* = 42), and atheist women (*n* = 38) in the control condition.

The three-way rmANCOVA Time × Condition × Group for the subscale *negative affective states* also failed to reach significance, Wilks’ λ = 0.997, *F*(2,223) = 0.28, *p* = 0.756, η_p_*^2^* = 0.00. The three groups did not significantly differ regarding their pre-exposure scores. However, the interaction Time × Condition, *F*(1,223) = 5.324, *p* = 0.022, η_p_*^2^* = 0.02, turned out to be significant, with subsequent *post hoc* tests indicating that negative affect did not change in the experimental condition (see Figure 3), *p* = 0.611, *d* = 0.05, but declined in the control condition (see Figure 4), *p* = 0.007, *d* = 0.26. Furthermore, the interaction Group × Condition, *F*(2,223) = 3.361, *p* = 0.036, *d* = 0.03, reached statistical significance. *Post hoc* tests revealed that compared to the control condition, Christian women reported greater negative affect in the experimental condition, *p* = 0.043, *d* = 0.43. In the additional analyses regarding the merged group of veiled and unveiled Muslim women, compared to the experimental condition, the merged group of veiled and unveiled Muslim women in the control condition reported greater negative affect, *p* = 0.032, *d* = 0.48. Participants’ means are shown in Table 3.

#### Emotions Toward the Models

The results of the univariate analyses revealed no significant group differences regarding the emotions toward the presented models in the experimental condition (see Table 2).

#### Correlations Between Pre-to-Post Body Image States Scale Score and Various Trait Measures

In the experimental condition, significant negative correlations emerged between the pre-to-post Body Image States Scale score and the Centrality of Religiosity Scale, the Eating Disorder Examination Questionnaire, the Eating Disorder Inventory 2 subscales, the Sociocultural Attitudes Toward Appearance Questionnaire, and admiration as well as rage toward the models (see Table 4). The more religious a woman was and the more she admired the models presented, the more her state body satisfaction decreased after being exposed to thin-ideal images. Furthermore, higher levels of disordered eating, drive for thinness, thin-ideal internalization, and pressure to be thin were positively correlated with a greater decrease in state body satisfaction in the experimental group. In contrast, the pre-to-post Body Image States Scale score negatively correlated with disgust toward the presented models. In the additional analyses regarding the merged group of veiled and unveiled Muslim women, the correlation between the pre-to-post Body Image States Scale score and the Centrality of Religiosity Scale score did not reach significance. However, a significant correlation was found between the pre-to-post Body Image States Scale score and the level of bulimia symptoms, *r* = −0.195, *p* = 0.030. Thus, the more bulimia symptoms a woman reported, the more her state body satisfaction decreased after being exposed to thin-ideal images.

**Table 4:** Pearson product-moment correlation coefficients between the pre-to-post Body Image States Scale score and the Centrality of Religiosity Scale, the Eating Disorder Examination Questionnaire total score, the Eating Disorder Inventory 2 subscales, the Sociocultural Attitudes Toward Appearance Questionnaire, the Physical Appearance Comparison Scale, and the emotions toward the models in the experimental condition (*N* = 118).

Variables	Pre-to-post Body Image States Scale score	
	*r*	*P*
Centrality of Religiosity Scale	−0.192^∗^	0.037
Eating Disorder Examination Questionnaire total score	−0.376^∗∗^	<0.001
Drive for thinness	−0.337^∗∗^	<0.001
Bulimia	−0.173	0.061
Body dissatisfaction	−0.370^∗∗^	<0.001
*Sociocultural attitudes toward appearance questionnaire*		
Internalization	−0.283^∗∗^	0.002
Pressure	−0.394^∗∗^	<0.001
Awareness	−0.118	0.205
Physical Appearance Comparison Scale	−0.154	0.097
*Emotions toward the models*		
Admiration	−0.367^∗∗^	<0.001
Rage	−0.221^∗^	0.017
Contempt	0.060	0.520
Disgust	0.183^∗^	0.049

*Positive pre-to-post Body Image States Scale scores indicate an increase in body satisfaction. ^∗∗^p < 0.001; ^∗^p < 0.05.*

## Discussion

The current study is the first to experimentally investigate whether veiled Muslim women’s state body image is affected by thin media images. The results indicate that although veiled Muslim women had a more positive trait body image than did Christian women and atheist women, the groups did not differ in terms of restraint eating, eating concerns, and drive for thinness. Furthermore, no differences between the three groups were found regarding changes in state body image from pre- to post-exposure. However, before and after the exposure in the experimental group, veiled Muslim women reported a more positive state body image than did Christian women. In terms of the findings of the additional analyses regarding the merged group of veiled and unveiled Muslim women, it is important to note that affiliation to Islam might be more decisive for a positive trait body image than veiling.

Overall, the first hypothesis, which stated that veiled Muslim women would report a more positive trait body image compared to Christian women and atheist women, was supported by the findings. However, the hypothesis assuming that veiled Muslim women would show less body dissatisfaction than Christian women and atheist women was not confirmed, as body dissatisfaction did not differ significantly between the three groups. In accordance with previous comparisons between veiled Muslim women and non-Muslim women (Đurović et al., 2016), in the present study, veiled Muslim women reported lower levels of internalization of the thin ideal and of pressure to conform to this ideal than did Christian women and atheist women. Thus, the results support the hypotheses that compared to Christian women and atheist women, veiled Muslim women would report a lower thin-ideal internalization and lower levels of pressure to be thin. However, as no significant differences were found regarding the awareness of the body ideal, it might be concluded that veiled Muslim women are aware of the Western beauty ideal, but do not put themselves under pressure to adhere to it. In support of this assumption, veiled Muslim women had a higher BMI compared to Christian and atheist women, but reported fewer shape concerns. Across the three groups, mean scores on the Sociocultural Attitudes Toward Appearance Questionnaire were similar to those from a German validation group (Knauss et al., 2009). Furthermore, in the current study, veiled Muslim women reported fewer physical appearance comparisons than did Christian women and atheist women, confirming the respective hypothesis. Compared to a non-clinical German validation group, Christian women and atheist women reported similar levels of physical appearance comparisons, whereas veiled Muslim women reported below-average levels (Mölbert et al., 2017). Given the negative correlation between hijab index and frequency of appearance comparisons, the veiling possibly prevented them from comparing their appearance with that of other women. The lower frequency of physical appearance comparisons might be one reason for the more positive body image of veiled Muslim women, as previous research stated that the extent of physical appearance comparisons correlates with body dissatisfaction (Myers and Crowther, 2009; Myers et al., 2012).

Moreover, the more frequently and the more conservatively Muslim women covered their bodies in the present study, the less they internalized and were aware of the thin ideal, supporting previous findings (Swami et al., 2014; Kertechian and Swami, 2017). However, in line with recent findings (Kertechian and Swami, 2017), the negative correlation with perceived pressure from the media did not reach significance. In addition, also confirming previous results (Swami et al., 2014; Wilhelm et al., 2018), no correlations were found between hijab index and participants’ BMI, body dissatisfaction, drive for thinness, as well as eating, weight, and shape concerns. These results suggest that although a more frequent and more conservative body covering is linked to some aspects of a positive trait body image, for instance less internalization of the thin ideal, other aspects of body image do not correlate with frequency and conservatism of veiling (i.e., body dissatisfaction). Moreover, in the present study, no relationship emerged between veiling and pressure to be thin, which is in contrast to previous research (Swami et al., 2014) and might be due to the different samples. Thus, a more frequent or conservative veiling might not protect Muslim women against pressure from the media. Instead, the resistance against Western ideals of beauty and the conscious decision to cover one’s body to protect it against the view and evaluations of strangers might lead to the more positive trait body image in veiled Muslim women. Given the non-significant correlations between age at beginning to veil and various trait body image measures, the age at which a woman started to veil and how she covers her body might not be relevant for a positive body image. However, it is of interest that the younger a woman was when she started to veil, the less she compared her body to those of others. A possible reason for this relationship might be that girls who begin to veil at a younger age become accustomed to not focusing on other women’s bodies, as they themselves do not want other people to see their own veiled body parts. Future research should investigate the reasons for this relationship and examine why veiled Muslim women seem to compare their shape to others to a lesser extent than do Christian women and atheist women.

It is important to note that the negative correlations between the hijab index and the internalization and awareness of the thin ideal, as well as the negative correlation between the hijab index and the level of physical appearance comparisons, disappeared when the merged group of veiled and unveiled Muslim women was included in the analysis. Therefore, it cannot be only the veiling *per se* that is related to a more positive trait body image. Rather, the affiliation to Islam, which might be associated with the more positive trait body image of the merged group of veiled and unveiled Muslim women in the current study, seems to be more decisive. Furthermore, given the positive relationship between body image and extent of religiosity (Akrawi et al., 2015), another reason for the more positive body image of the merged group of veiled and unveiled Muslim women compared to Christian women and atheist women might lie in the Muslim women’s higher levels of religiosity.

Regarding restraint eating and eating concerns, veiled Muslim women did not differ from Christian women and atheist women. This supports recent findings, which also indicated no group differences in disordered eating (Wilhelm et al., 2018). Furthermore, in line with previous results (Wilhelm et al., 2018), no group differences emerged regarding the subscales drive for thinness and bulimia symptoms. Although, compared to the other two groups, veiled Muslim women had lower levels of shape and weight concerns, thin-ideal internalization and pressure to be thin, they also reported an urge to be thin as well as restraint eating and eating concerns. Across all groups, the mean levels of eating, weight, and shape concerns were similar to those reported for a non-clinical German validation group (Hilbert and Tuschen-Caffier, 2016); the mean levels of drive for thinness and bulimia symptoms were slightly higher than in a non-clinical German validation group (Paul and Thiel, 2005). Regarding Body Dissatisfaction, the mean values of the three groups were similar to those of a non-clinical German validation group (Paul and Thiel, 2005). These findings suggest that, in veiled Muslim women, disordered eating is not necessarily associated with a negative body image, but might be related to biological, sociocultural, or other psychological factors. For example, a study in Muslim women from the United Arab Emirates found a positive correlation between extent of religiosity and eating disorder symptoms, and the authors discussed perfectionism as an underlying factor for both outcomes (Thomas et al., 2018).

The results from part 2 confirmed the second hypothesis, which stated that women would report a more negative state body image after watching a slide show of thin models compared to viewing pictures of furniture. In line with results of meta-analyses (Groesz et al., 2002; Grabe et al., 2008; Want, 2009), body satisfaction declined in the experimental condition and not in the control condition. In support of previous results (Groesz et al., 2002; Want, 2009), correlation analyses showed that the more body image concerns a woman reported prior to the experiment, the more her state body satisfaction declined from pre- to post-exposure. Interestingly, in the control condition, women reported lower levels of negative affect after the exposure to pictures of furniture compared to before the exposure. Discussions with the participants after the exposure revealed that many women were interested in interior design and therefore felt inspired by the pictures of furniture, which might have led to the decrease in negative affect.

The third hypothesis, which stated that after exposure to thin images, veiled Muslim women would report a more positive state body image compared to Christian and atheist women, was not confirmed by the findings. Although veiled Muslim women in the experimental condition reported a more positive state body image than did Christian women, no differences were found between veiled Muslim women and atheist women. In addition, body satisfaction decreased significantly from pre- to post-exposure across all groups in the experimental condition compared to the control condition. Thus, the veiling did not buffer veiled Muslim women against the negative effects of media. In line with this finding, veiled Muslim women did not differ from Christian and atheist women regarding their changes in negative affect from pre- to post-exposure. Therefore, in the present study, previous findings for non-Muslim women (Groesz et al., 2002; Grabe et al., 2008; Want, 2009) were confirmed for veiled Muslim women. Veiling did not buffer against the body-related threat, i.e., being exposed to the slide show of thin models, which is in line with the non-significant correlation between the hijab index and perceived pressure to be thin from the media. As a more conservative and frequent veiling was not linked to pressure from the media, it seems that veiling might not be related to effects of media on body image. Instead, being exposed to thin-ideal images negatively affects the state body image of women regardless of their extent of religiosity.

A possible explanation for the findings might lie in the clothing of the presented models. As women with higher religiosity, and especially veiled Muslim women, generally wear more modest clothing than do fewer religious women (Mussap, 2009; Lewis and Tarlo, 2011), veiled Muslim women might not have identified with the presented models wearing short and tight clothes. This lack of identification might have inhibited self-enhancement processes, leading to an increase in body satisfaction (Bessenoff, 2006). Furthermore, veiled Muslim women might have opposed the style of clothing of the presented models, as it does not comply with their clothing regulations, and might therefore have felt uncomfortable during the testing situation.

The latter point leads to the limitations of the current study, as we only used unveiled model pictures, which might have different effects on veiled Muslim women compared to Christian or atheist women. Furthermore, only Muslim women were asked about their clothing; thus, no information was gained about the dress style of the Christian and atheist women. A major limitation of the present study is the lack of a group of unveiled Muslim women. Future research should obtain an adequate number of unveiled Muslim women participants, as direct comparisons between veiled and unveiled Muslim women would allow for conclusions about the effect of religion and veiling on body image. A further limitation is that the sample was heterogeneous in many respects, e.g., culture of origin. Moreover, participants’ use of social media was not assessed. Given that social media is becoming increasingly important in body image research (Perloff, 2014; Fardouly and Vartanian, 2016; Wagner et al., 2016), future studies might investigate the potentially differing use of social media in these groups. Another limitation is that participants’ relationship to God was not specified, for instance participants did not report whether they feel accepted by God or whether their religious motivation is intrinsic or extrinsic. In addition, there was no differentiation between the various types of Islam or of Christianity. Furthermore, the study did not include any measures assessing commitment, although general commitment might be one crucial factor for a positive body image (Inman et al., 2014). Lastly, no cover story was used in the current study and participants were not asked whether they guessed the aim of the experiment, which could have influenced the results. On the other hand, a strength of the study is the first use of an experimental approach in order to investigate how veiling might affect Muslim women’s state body image. Another strength of the study lies in the separate, highly differentiated investigation of veiled Muslim women’s trait body image.

In sum, veiled Muslim women had a more positive trait body image compared to Christian women and atheist women; for instance, they reported lower levels of thin-ideal internalization and of pressure to be thin. However, the three groups did not differ regarding restraint eating, eating concerns, and bulimia symptoms. Moreover, compared to women who were exposed to pictures of furniture, women who were exposed to pictures of thin and attractive models showed a decline in state body satisfaction. Accordingly, being exposed to thin media images negatively affected women’s state body image irrespective of religiosity and veiling. However, veiled Muslim women in the experimental condition reported a more positive state body image than did Christian women before and after the exposure. The results of the additional analyses regarding the merged group of veiled and unveiled Muslim women showed that the merged group of veiled and unveiled Muslim women reported a more positive trait body image compared to Christian women and atheist women. Moreover, when the merged group of veiled and unveiled Muslim women was included in the analysis, the negative correlations between the hijab index and the internalization and awareness of the thin ideal, as well as the negative correlation between the hijab index and the level of physical appearance comparisons, disappeared.

The present findings give rise to the following theoretical and practical implications and recommendations. Given that restraint eating and dieting are key factors for the development of eating disorders (Stice and Shaw, 2002) and that the latter have highly negative effects on health and quality of life (Stice and Shaw, 2002; Carta et al., 2014), it is important that programs to prevent disordered eating also address veiled Muslim women. Furthermore, practicing physicians, psychologists, and psychotherapists should be trained to detect and to treat eating disorders in veiled Muslim women, and it might also be advisable for them to strengthen their intercultural skills and their cultural sensitivity. Regarding the negative effects of thin-ideal media on state body image, it seems necessary to convey a critical approach to mass media. For example, it might be important to state that pictures presented in the media or on social media platforms are often edited using image manipulation programs (Sarvas and Frohlich, 2011). Moreover, the presentation of very thin models in mass media and on social media should be critically discussed, for instance on the social media platforms themselves (Perloff, 2014). By posting pictures of normal-weight women on social media or by presenting normal-weight models in the mass media more often, it may become clearer that it is neither attainable nor desirable for everyone to be very thin (Perloff, 2014).

In terms of theoretical implications, it would be interesting to examine why the groups did not differ regarding disordered eating, even though veiled Muslim women had a more positive trait body image compared to Christian women and atheist women. In this context, future studies should investigate the relationships among perfectionism, religiosity, and eating disorder symptoms in veiled Muslim women. Moreover, it would be useful to include models wearing loose-fitting clothes and a hijab as an experimental condition in order to test whether veiled Muslim women identify to a greater degree with models wearing some type of Islamic body covering than with models wearing Western clothing. Finally, future research should examine whether the state body image of veiled Muslim women might also be affected by other body-related threats, for instance the intake of food (Vocks et al., 2007).

## Ethics Statement

This study was carried out in accordance with the recommendations of the Ethics Committee of the University Osnabrück with written informed consent from all subjects. All subjects gave written informed consent in accordance with the Declaration of Helsinki. The protocol was approved by the Ethics Committee of the University Osnabrück.

## Author Contributions

LW, AH, JB, and SV: conceptualization. LW and MW: formal analysis. LW: investigation and writing – original draft. LW and SV: methodology. LW and MK: recruitment. SV: supervision. LW, AH, JB, MW, and SV: writing – review and editing.

## Conflict of Interest Statement

The authors declare that the research was conducted in the absence of any commercial or financial relationships that could be construed as a potential conflict of interest.
